# Pulmonary epithelioid hemangioendothelioma: A rare pulmonary tumor in differential diagnosis of bronchogenic carcinoma

**DOI:** 10.4103/0970-2113.59268

**Published:** 2010

**Authors:** Anshuman Darbari, Devender Singh, Prashat Kumar Singh, Manu Bharadwaj

**Affiliations:** C.T.V.S. Department, Wokhardt Hospital, Surat - 395 001, Gujrat, India

Sir,

A 33-year-old female was admitted to our institute with complaints of dry cough with minimal expectoration and two episodes of hemoptysis in the last four years. She was also complaining of intermittent chest pain and loss of appetite but without any weight loss for last four months. She had no prior medical history, was a non-smoker, and denied any history of exposure to pets or birds. There was no other family history of cancer. She was average built and, on physical examination, showed no clubbing of fingers and toes, no enlarged lymph nodes. Lung examination revealed minimal inspiratory crackles at the left base without any added sounds or evidence of consolidation.

**Figure 1:**
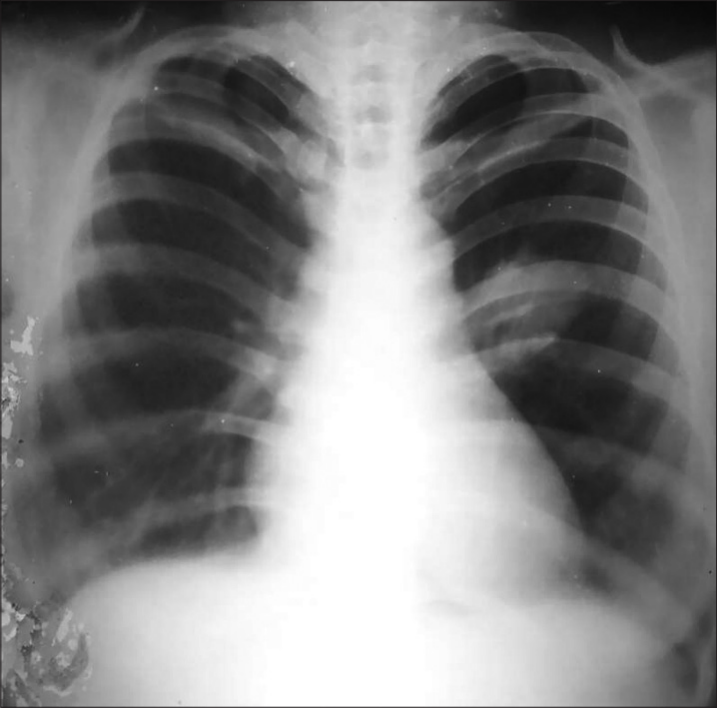
Chest radiograph shows a rounded mass lesion in the left hemithorax without any associated bony abnormality

**Figure 2:**
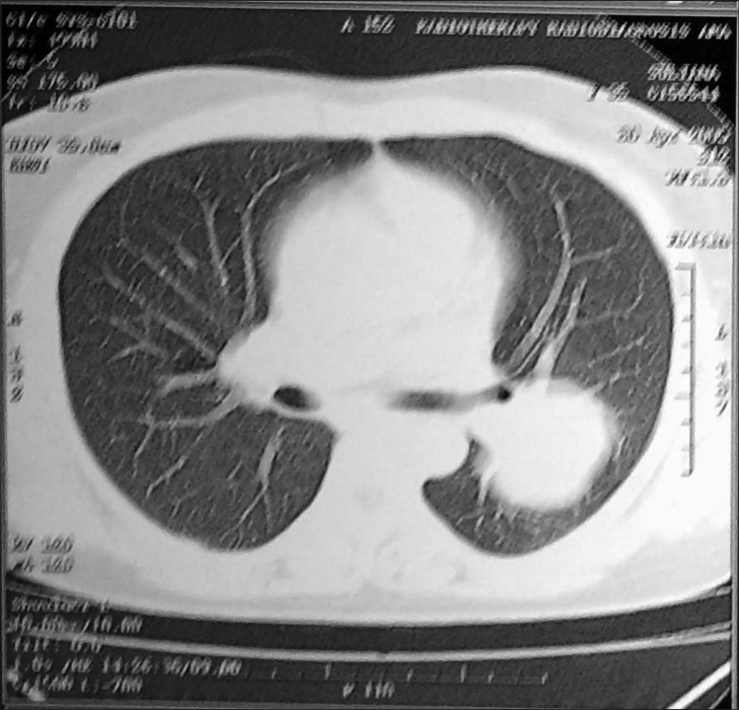
Computed tomography (CT) scan shows a well defined mass in the left lung; there was no hilar or mediastinal lymphadenopathy in the mediastinal windows

Routine blood investigations were in normal limits. Sputum smears were negative for acid fast bacilli, fungal elements and malignant cells. Chest radiograph showed about 6 × 6 cms round opacity in mid zone of left lung [Figure 1]. Contrast enhanced computerized tomographic (CT) scan of thorax showed heterogeneously enhancing soft tissue density mass lesion in apical segment of left lower lobe [Figure 2]. CT guided needle aspiration cytology of mass showed atypical epithelial cells along with keratinous material and was suspicious of squamous cell carcinoma. Ultrasound abdomen was normal. No endobronchial lesions were found at fiber optic bronchoscopy. Pulmonary function tests were within normal range. Under general anesthesia, we found a 7 × 7 cms firm vascular mass, of heterogeneous consistency, cystic in between and involving major fissure with both lobes of left lung without any pleural and vascular invasion. Left sided extra pericardial pneumonectomy was done. Postoperative course was uneventful and she was discharged after a week in satisfactory condition. Biopsy of specimen diagnosed it as epithelioid hemangioendothelioma of lung. Later, immuno-histochemistry was done for confirmation of the diagnosis, vascular tumor markers CD31 (CD = cluster of differentiation- antigen class) and CD34 (CD = cluster of differentiation- antigen class) were positive. Patient survived without any morbidity and is doing well so far (postoperative three years). Due to asymptomatic nature, no additional imaging modalities were done.

The tumor was initially described in 1975 by Dail and Liebow and named “intravascular sclerosing bronchioalveolar tumor” (IVBAT) because of the proposed bronchioalveolar origin of the neoplasm. Enzinger and Weiss (1988) first used the term hemangioendothelioma. Now it is recognized that this is the pulmonary counterpart of epithelioid hemangioendothelioma, which is sclerosing true neoplasm of vascular endothelium of low grade to borderline malignancy potential and occurring around medium to large size veins in soft tissue, occurring also in other anatomical sites.[1–3]

Three quarters of patients with epithelioid hemangioendothelioma of the lung are women. The age range at presentation is wide (12 - 60 years). Half the patients are asymptomatic and are discovered on incidental chest radiography. Symptoms are uncommon and are usually include dyspnea, mild pleuritic pain, non-productive cough and rarely hemoptysis. The chest radiograph usually reveals multiple or bilateral pulmonary nodules. Pleural effusions and distant metastases are rarely seen.[1–3]

Histology is diagnostic for these tumors.[1,3] The characteristic immunophenotype is negative for epithelial, muscular, and neural markers, and positive for vascular markers such as factor VIII, CD31 (Most specific and sensitive endothelial marker), CD34, von Willebrand's factor, and Ulex europaeus antigen (UEA-1). Ultra-structural endothelial characteristics can be confirmed by electron microscopy, which shows the presence of prominent basal lamina, pinocytotic vesicles, and Weibel-Palade bodies.[1,3,4]

Because of the rarity of this condition, there is no clear standard for treatment. In most cases, the disease has a slowly progressive course. Apparently, benign lesions of hemangioendothelioma are best treated by wide local excision and apparently malignant ones by radical excision.[1,3,4]

Epithelioid hemangioendothelioma is a rare pulmonary neoplasm of vascular origin with fewer than 50 cases reported in the literature.[3,5] We report one such case with an initial presentation mimicking bronchogenic carcinoma but proven to be pulmonary epithelioid hemangioendothelioma on histopathological and immunohistochemical examination of the surgically resected tumor. It is also unusual because of the solitary nature.
